# FOOD INSECURITY AMONG OLDER ADULTS IN THE UNITED STATES DURING THE COVID-19 PANDEMIC

**DOI:** 10.1093/geroni/igad104.0289

**Published:** 2023-12-21

**Authors:** Naglaa El-Abbadi, Zhongqi Fan, Amy Yang, Ana Ronan, Ryan simpson, Kimberly Nguyen, Elena Naumova

**Affiliations:** Jean Mayer USDA Human Nutrition Research Center on Aging, at Tufts University, Boston, Massachusetts, United States; MIT, Cambridge, Massachusetts, United States; Tufts University, Boston, Massachusetts, United States; Arizona State University, Glendale, Arizona, United States; Tufts University, Boston, Massachusetts, United States; Tufts University, Boston, Massachusetts, United States; Tufts University, Boston, Massachusetts, United States

## Abstract

The COVID-19 pandemic severely impacted food access and nutritional security across the United States, and exacerbated disparities in household food insecurity rates particularly among older adults due to potential for reduced food access, mental or physical health limitations, or fixed incomes. This study investigated food insecurity among older adults in the US throughout time periods of the pandemic, exploring differences among population groups for food insecurity risk factors. Data was from the US Census Bureau’s Household Pulse Survey, April 2020 through December 2022, weight-adjusted for a nationally representative sample (n=3,884,922). Logistic regression models were constructed to examine the association between older age and self-reported household food insecurity. Reasons given for food insecurity were examined as secondary outcomes. Covariates included demographics, socioeconomic status, household structure, food support programs, and mental health status. Findings showed risk of food insecurity among adults aged 60+ years more than doubled among minority racial groups compared to non-Hispanic White (p<0.001) but was inversely associated with being female compared to male (OR 0.77, p<0.001). Older adults who reported experiencing at least one mental health indicator (anxiety, depression, low interest, or worry) more than half of the week had over five times the odds of being food insecure (OR 5.68, p<0.001). Overall, trends and factors associated with prevalence and reasons for food insecurity were found to vary across age groups, and at different points of the pandemic. Our findings can help elucidate factors to identify older adult groups at greater food insecurity risk.

